# Anticancer Properties of PPAR*α*-Effects on Cellular Metabolism and Inflammation

**DOI:** 10.1155/2008/930705

**Published:** 2008-05-20

**Authors:** Maja Grabacka, Krzysztof Reiss

**Affiliations:** ^1^Department of Food Biotechnology, Faculty of Food Technology, Agricultural University of Krakow, ul. Balicka 122, 31149 Krakow, Poland; ^2^Department of Neuroscience, Center for Neurovirology, School of Medicine, Temple University, Philadelphia, PA 19140, USA

## Abstract

Peroxisome proliferator-activated receptors (PPARs) have lately attracted much attention as therapeutic targets. Previously, PPAR ligands were associated with the treatment of diabetes, hyperlipidemia and cardiovascular diseases, as they modulate the expression of genes regulating glucose and lipid metabolism. Recently, PPAR ligands have been also considered as potential anticancer agents, with relatively low systemic toxicity. The emerging evidence for antiproliferative, proapoptotic, antiinflammatory and potential antimetastatic properties of PPAR*α* ligands prompted us to discuss possible roles of PPAR*α* in tumor suppression. PPAR*α* activation can target cancer cells energy balance by blocking fatty acid synthesis and by promoting fatty acid *β*-oxidation. In the state of limited nutrient availability, frequently presents in the tumor microenvironment, PPAR*α* cooperates with AMP-dependent protein kinase in: (i) repressing oncogenic Akt activity, (ii) inhibiting cell proliferation, and (iii) forcing glycolysis-dependent cancer cells into “metabolic catastrophe.” Other potential anticancer effects of PPAR*α* include suppression of inflammation, and upregulation of uncoupling proteins (UCPs), which attenuates mitochondrial reactive oxygen species production and cell proliferation. In conclusion, there are strong premises that the low-toxic and well-tolerated PPAR ligands should be considered as new therapeutic agents to fight disseminating cancer, which represents the major challenge for modern medicine and basic research.

## 1. PPAR*α* AND CANCER CELL ENERGY BALANCE

The concept that neoplastic transformation based on the failure of energy homeostasis is currently regaining considerable interest. This notion was associated with the hypothesis by Otto Warburg who indicated a distinctive dependence of tumor cell metabolism from glycolysis, even when there is sufficient amount of oxygen available for much more efficient oxidative phosphorylation [1, 2]. Only recently, it has been established that the inclination of tumor cells for glycolysis is mainly driven by mitochondrial dysfunction or oncogenic activity of Akt, Ras, or Myc [3, 4].

PPAR*α*, which is a transcriptional activator of fatty acid *β*-oxidation machinery (e.g., acyl-CoA oxidase (ACO), acyl-CoA synthetase (ACS), carnitine palmitoyl transferase (CPT1), fatty acid binding protein (FABP), and fatty acid transporter (FAT)), can switch energy metabolism toward fatty acid degradation, and decrease glucose uptake by repressing glucose transporter GLUT4 [5, 6]. Interestingly, PPAR*α* acts as a direct sensor for fatty acids, which are considered natural ligands for this nuclear receptor [7, 8]. According to fatty acid, glucose cycle paradigm increased rate of fatty acid and ketone bodies oxidation forces the decline in glucose utilization through the inhibition of glycolytic enzymes [9, 10]. This concept was supported by the results of animal studies, showing that during fasting-activated PPAR*α* can divert energy metabolism from the glucose to fatty acid utilization as a primary source of energy.

Mitochondria are the main organelles that carry out fatty acid *β*-oxidation and produce ATP through oxidative phosphorylation [11]. Oncogenic transformation is frequently associated with mitochondrial dysfunction, however, it is still controversial if this is a result, cause, or contribution to the malignant phenotype [12]. A direct link between aerobic respiration and carcinogenesis has been provided by the demonstration that the loss of p53, which is most commonly mutated gene in cancer, results in decrease of synthesis of cytochrome c oxidase (SCO2) gene expression [13]. SCO2 is crucial for the incorporation of mitochondrial DNA-encoded cytochrome c oxidase subunit (MTCO2) into the cytochrome c oxidase complex, and the proper assembly of this complex is essential for the mitochondrial respiration. Therefore, SCO2 downregulation in p53-deficient cells heavily inpairs oxidative phosphorylation and triggers the switch toward glycolysis [13].

Furthermore, loss of function mutations in the nuclear genes encoding the Krebs cycle enzymes (such as succinate dehydrogenase and fumarate hydratase) are frequently observed in uterine leiomyomas, renal carcinomas paragangliomas, and phaeochromocytomas [14]. The clinical data suggest that these proteins might have other functions besides energy metabolism and can be involved in the induction of apoptosis, similarly to mitochondrial apoptosis inducing factor (AIF) [15]. Nevertheless, it is likely that the glycolysis-promoting metabolism of cancer cells relieves the selection pressure and permits clonal growth of the cells with defective mitochondrial system. Such cells could be brought to the verge of metabolic catastrophe in the condition of limited glucose availability or when the oxidative metabolism is forced pharmacologically. This opens an opportunity for the use of PPAR*α* ligands, as they should be selectively toxic for cancer cells and neutral for normal cells.

Energetic function of mitochondria is not restricted to ATP generation in the process of oxidative phosphorylation. Systemic thermal homeostasis maintained by mammals relies broadly on nonshivering thermogenesis carried on by brown adipocytes. In these cells, uncoupling protein (UCP1) is responsible for the “proton leak” of mitochondrial inner membrane, which separates respiration from ATP synthesis. The energy released through the proton flow in line with electric potential gradient is dissipated as heat.

Recently, several mammalian UCP homologues have been discovered, among which ubiquitously expressed UCP2 and muscle—specific UCP3 gained deep interest [16]. They share high degree of structural similarity with UCP1 though their primary function, which still remains elusive, is not limited to thermogenesis, but their mitochondrial uncoupling activity is connected with fatty acid anion transport. The expression of both UCP2 and UCP3 is regulated by PPAR*α* [6, 17–19], and this notion provides an interesting link with cancer cell metabolism and behavior.

The recent report by Pecqueur and colleagues [20] has revealed that UCP2 controls proliferation through driving cellular metabolism to fatty acid oxidation and limiting glycolysis. UCP2- deficient cells proliferate significantly faster than wild-type cells and rely on glycolysis-derived pyruvate catabolism, like all rapidly normal and transformed dividing cells do. Remarkably, the higher proliferation rate in these cells is a result of cell cycle shrinkage and not the decrease in the quiescent (G0/G1) cell fraction, even though the proproliferative PI3K/Akt and MAPK signaling pathways are more activated in UCP −/− than wt cells [20]. Interestingly, UCP2 is also involved in cellular adhesion and invasive potential, as was revealed in the studies on the THP1 monocytes with UCP2 overexpression, which showed impaired *β*2 integrin—mediated adhesion and transendothelial migration [21]. Taking together, these data suggest that PPAR*α*-mediated UCP2 upregulation might have a negative impact on cancer progression.

Uncoupling proteins due to their ability to reduce ATP bisynthesis inhibit production of reactive oxygen species (ROS) during respiration. ROS and products of their activity, such as lipid peroxides, are not only toxic and mutagenic, but also stimulate inflammatory response, and therefore contribute to cancer development. PPAR*α* regulates the expression of three proteins which govern the transport of fatty acids in and out of mitochondria. This includes CPT1 and UCP3 as well as mitochondrial thioesterase 1 (MTE-1) [17, 22]. This trio controls the mitochondrial pool of fatty acids in order to keep the danger of their peroxidation at minimal level. CPT1 supplies mitochondria with long chain fatty acid—CoA (LCFA-CoA) complexes, which undergo *β*-oxidation. At a high rate of *β*-oxidation, UCP3 in the conjunction with MTE-1 acts to prevent LCFA-CoA accumulation: MTE-1 releazes CoA-SH and enables its recycling, whereas UCP3 exports fatty acid anions outside the mitochondrial matrix, and therefore reduces the chance of their peroxidation by the superoxide generated in the complex I and III of mitochondrial electron chain [23–25]. Simultaneously, UCP2 and UCP3 due to their proton leak activity reduce the rate of ROS production, which is proportional to the protonmotive force [16, 26]. The hypothesis of protective role of PPAR*α* in oxidative stress is supported by the results from in vivo studies showing that PPAR*α*-deficient mice have higher level of oxidative damage in cardiac muscle, and that fenofibrate diminishes inflammatory response and oxidative stress in the neural tissue in rats subjected to traumatic brain injury [27, 28].

The above described evidence indicates that PPAR*α* activation might metabolically target neoplastic cells through inhibition of glycolysis and promotion of fatty acid catabolism, but also might elicit chemopreventive effect through the decrease of respiratory ROS production.

Interestingly, the metabolic peculiarities of cancer cells are not restricted to aerobic glycolysis but paradoxically include also fatty acid synthesis. Some types of tumors, particularly of hormone responsive epithelial origin, are characterized by the abnormally high activity of fatty acid synthase (FAS), which is an enzyme with barely detectable levels in normal tissues. The FAS produces palmitate from the condensation of acetyl-CoA and malonyl-CoA. Interestingly, FAS overexpression correlates well with prostate cancer progression in which the highest levels of FAS activity have been observed in bone metastases [30]. For this reason, FAS has been named a “metabolic oncogene” [31]. FAS is also involved in biosynthesis of phospholipids, which are substrates for the new membrane synthesis in rapidly dividing cells, protein myristoylation, and lipid partitioning into membrane microdomains [31, 32]. FAS activity provides a significant growth advantage for transformed cells. Indeed, pharmacological inhibition of FAS induced apoptosis in cancer cell, possibly by the accumulation of malonyl-CoA [33]. In addition, pharmacological or RNA silencing-mediated inhibition of FAS significantly reduced the expression of the oncogenic Her-2/neu (erbB-2) [34, 35], but it also induced a dramatic increase in VEGF expression by activating the Erk1/2 pathway [36].

**Figure 1:**
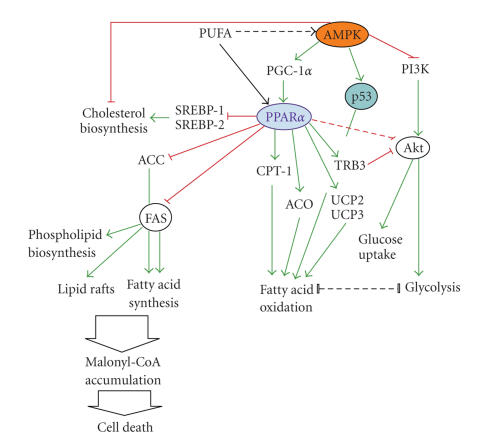
PPAR*α* interferes with the metabolic pathways in the cancer cells. In the state of energy deprivation, AMPK activates fatty acid oxidation through PPAR*α*- and p53-dependent pathways and blocks anabolic processes, for example, cholesterol biosynthesis. AMPK is a potent inhibitor of Akt-induced glycolysis. In response to nutrient deficiency, PGC-1*α* and PPAR*α* upregulate expression of TRB3, which inactivates Akt via direct interaction [29]. PPAR*α* promotes fatty acid *β*-oxidation as a transcriptional activator of fatty acid catabolic enzymes and transport proteins (e.g., ACO, CPT1, UCP2, and UCP3). Simulateneously, PPAR*α* blocks lipid synthesis by repression of SREBP-1 and -2, ACC, and FAS. FAS inhibition in various cancer types results in toxic accumulation of malonyl-CoA and apoptosis. For more details, see the text. Arrowheads represent activation/upregulation, and blunted lines indicate inhibition/downregulation of the cellular proteins or processes. ACC—acetyl-coA carboxylase; ACO—acyl-coA oxidase; AMPK—AMP-dependent kinase; CTP-1—carnitine palmitoyltransferase-1; FAS—fatty acid synthase; PGC-1*α*—PPAR*γ* coactivator 1*α*; PUFA—polyunsaturated fatty acids; SREBP—steroid response element binding protein; TRB3—mammalian homolog of *tribbles*; UCP2, UCP3—uncoupling proteins.

Importantly, activation of PPAR*α* has been shown to block FAS pathways through the transcriptional repression of genes, which are directly involved in its metabolic activity (FAS; acyl-CoA carboxylase (ACC); steroid response element binding proteins (SREBP1, SREBP2)) [37–41] (Figure 1). Simultaneously, PPAR*α* blocks Erk1/2 activation [42]. Therefore, the possibility exists that PPAR*α* agonists could block Her-2/neu expression without a danger of proangiogenic stimulation of VEGF expression. This might encourage new clinical applications for PPAR*α* ligands against those cancer cells, which are characterized by the overactive FAS.

Lipid metabolism deregulation manifested by hiperlipidemia has been described as a significant risk factor for colorectal cancer development [43]. Increased serum trigliceride and cholesterol level were observed in patients with familial adenomatous polyposis coli. An interesting study by Niho and coworkers [44] showed that APC-deficient mice, the animal model for human adenomatous polyposis coli syndrome, when treated with PPAR*α* ligand and lipid level normalizing drug—bezafibrate, develop significantly fewer intestinal polyps. This protective action of PPAR*α* agonists against colorectal carcinogenesis seems promising from the therapeutic point of view, suggesting that the patients might benefit not only from normolipidemic activity of PPAR*α*, but also from its antineoplastic effects as well.

## 2. AMPK AND AUTOPHAGY

**Figure 2:**
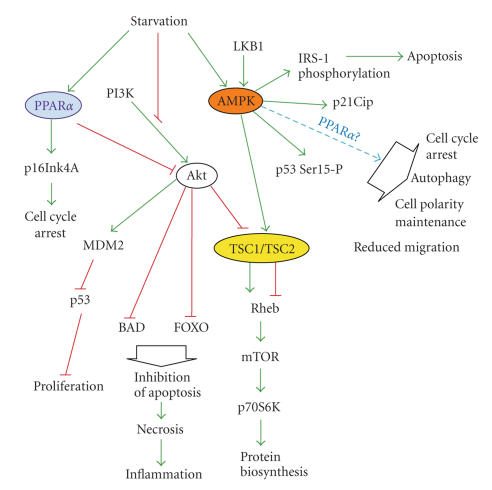
PPAR*α* and AMPK activities in the cancer cells exposed to energetic stress. AMPK switches on p53-dependent cell cycle metabolic check point and autophagy and blocks Akt/mTOR protein de novo synthesis pathway. PPAR*α* induces cell cycle arrest and downregulates Akt neutralizing its antiapoptotic actions. For more details, see the text. Arrowheads represent activation/upregulation, and blunted lines indicate inhibition/downregulation of the cellular proteins or processes. IRS-1—insulin receptor substrate-1; mTOR—mammalian target of rpamycin kinase; TSC1—tuberous sclerosis 1 (hamartin); TSC2—tuberous sclerosis 2 (tuberin).

In the state of energy depletion, caused for instance by a limited glucose availability, normal cells can switch between energy metabolic pathways to support their survival. AMP-dependent protein kinase (AMPK) plays an integral role in the response to starvation by sensing the rise in AMP/ATP ratio and switching off the ATP-consuming anabolic processes, such as protein and lipid synthesis or DNA replication. AMPK can induce several rescue pathways, which enhance cell survival during glucose deprivation (Figures 1 and 2). One of them includes p53-dependent check point, which blocks cell cycle progression and promotes fatty acid oxidation and autophagy, as an alternative source of energy [45, 46]. Interestingly, p53-deficient cancer cells are very sensitive to the lack of glucose, and being incapable of autophagy, underwent massive apoptosis [46, 47]. It was demonstrated that PPAR*α* acts downstream from AMPK and was responsible for AMPK-induced fatty acid oxidation in cardiac and skeletal muscle [48, 49]. This might suggest that PPAR*α* mediates other activities of AMPK. AMPK is a potent inhibitor of PI3K/Akt signaling, especially of Akt-induced glycolysis and protein synthesis [45, 50, 51]. Oncogenic Akt is responsible for increased activity of mammalian target of rapamycin (mTOR) kinase, which phosphorylates downstream regulators of translation such as 4EBP-1 and p70S6 kinase (Rsk) [51, 52]. AMPK antagonizes this Akt-induced mTOR activation by activating tumor suppressor tuberous sclerosis 2 (TSC2, tuberin), which in turn inactivates a small G-protein, Rheb, and in consequence disabled Rheb cannot activate mTOR [53–55]. Some of theses multiple signaling and metabolic connections between PPAR*α*, AMPK, and mTOR are additionally explained in Figures 1 and 2.

We have demonstrated that PPAR*α* activation inhibits Akt phosphorylation and reduces the metastatic potential of mouse melanoma cells [42]. This may provide an interesting synergy between AMPK and PPAR*α* toward mTOR inhibition and the activation of autophagy. Although the mechanism by which fenofibrate attenuates Akt phosphorylation is still under investigation. It has recently been reported that fenofibrate increases plasma membrane rigidity in a manner similar to elevated cholesterol content [56]. In this report, fenofibrate did not change the membrane content of cholesterol but increased plasma membrane rigidity by itself, altering activities of different membrane-bound proteins. Therefore, one could speculate that fenofibrate, besides its role as a PPAR*α* agonist, may also act in a nonspecific manner by altering membrane-bound growth factor receptors such as IGF-IR or EGFR, which are known to have a strong signaling connection to Akt. Further experiments are required to determine whether similar fenofibrate-mediated changes in the fluidity of the plasma membrane are indeed responsible for the attenuation of the ligand-induced clustering of receptor molecules—a critical step in the initiation of growth and survival promoting signaling cascades.

It has also been demonstrated that omega 3 polyunsaturated fatty acids (n-3 PUFA), which are potent ligands of PPAR*α*, induce fatty acid *β*-oxidation via AMPK [57]. AMPK is regulated by a tumor suppressor LKB1 and coordinates various cellular responses, which can exert antineoplastic effects [58]. One of them is autophagy, which has been intensively explored in the context of carcinogenesis. Autophagy, also called a type II programmed cell death, is a lysosomal-mediated digestion of different cellular components, including organelles to obtain energy, however, it may also lead to cell death [3, 59, 60]. There is a growing body of evidence that defective autophagy may result in cancer progression [59, 61]. Beclin 1, a protein required for autophagy, is frequently lost in ovarian, breast, and prostate cancers, and beclin 1 +/− mutant mice are prone to increase incidence of tumors derived from epithelial or lymphopoietic tissues [62, 63]. Autophagy is negatively controlled by Akt/PI3K signaling and specifically by mTOR, which acts as a sensor of growth stimuli and nutrient availability and at the same time is the main target for the rapamycin-mediated antitumor activity [64]. Degenhardt et al. [3] demonstrated that cells transformed by Akt overexpression and by deficiency in proapoptotic genes, BAX, and BAK show a highly invasive phenotype, however, became necrotic when deprived of oxygen and glucose.

Although Akt activation provides a growth advantage, it simultaneously impairs autophagy in response to metabolic stress and condemns cells to necrotic death. Abundant necrosis stimulates inflammation and enhances macrophage infiltration within tumors, which is a poor prognostic factor, and actually accelerates tumor growth [3]. These findings support the notion that loss of autophagy in apoptosis-incompetent cells can have tumor promoting effects. This can happen in cells with constitutively activated Akt, as it triggers a strong antiapoptotic signal, mainly by the inactivation of proapoptotic proteins, BAD, and FOXO [52].

In the state of nutrient deprivation, AMPK induces autophagy in a p53-dependent manner and evokes apoptosis through the serine phosphorylation of insulin receptor substrate (IRS-1), which in turn inhibits PI3K/Akt signaling pathway [65]. It is not known if PPAR*α* is involved in these actions downstream of AMPK, but possibly can support them by the inhibition of Akt [66]. In this respect, inhibition of Akt by PPAR*α* ligand, fenofibrate, significantly suppressed anchorage-independent growth, cell motility and cell migration in vitro; and in the experimental animal model, fenofibrate treatment reduced metastatic spread of hamster melanoma cells to the lungs [42, 67]. This apparent inhibition of cell migration and compromised cell invasiveness was likely associated with alterations in the cytoskeletal structure. Interestingly, AMPK has been implicated in the maintenance of epithelial cell polarity, by affecting actin-fiber distribution during energy deprivation [68]. In particular, AMPK mutations disrupted the polarity of the epithelium and triggered tumor-like hyperplasia, again supporting the notion of a possible cooperation between PPAR*α* and AMPK.

## 3. PPAR*α* AND INFLAMMATION

**Figure 3:**
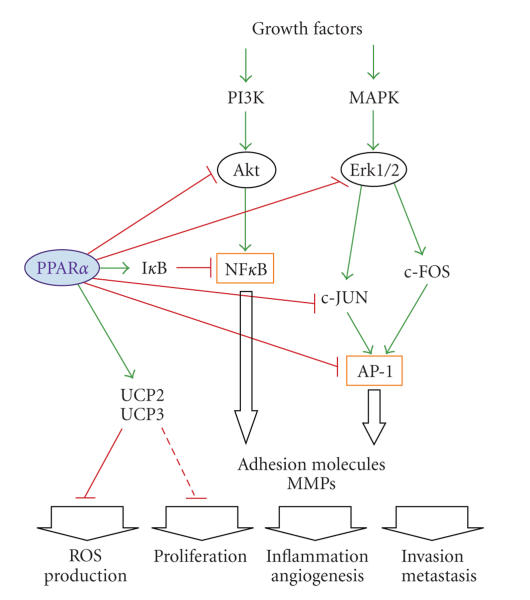
PPAR*α* antagonizes main inflammatory signaling pathways through repression of the main inflammatory transcription factors: NF*κ*B and AP-1. Additionally, PPAR*α* reduces ROS-mediated inflammation by upregulation of uncoupling proteins UCP2 and UCP3. See the text for more detailed explanation. Arrowheads represent activation/upregulation, and blunted lines indicate inhibition/downregulation of the cellular proteins or processes. AP-1—activating protein-1; Erk1/2—extracellular signal response kinase 1/2; I*κ*B—inhibitor of NF*κ*B; MAPK—mitogen activated protein kinase; NF*κ*B—nuclear factor *κ*B; ROS—reactive oxygen species.

The anticancer effects of activated PPAR*α* can be attributed to its well-characterized anti-inflammatory properties. PPAR*α* inhibits expression of variety of inflammatory genes, such as interleukin 6 (IL-6) and inducible cyclooxygenase-2 (COX-2), as well as reduces nitric oxide production in murine macrophages exposed to bacterial lipopolisaccharide (LPS) [69–71]. These events can be ascribed to the PPAR*α* antagonistic action against the main transcription factors mediating inflammatory responses, nuclear factor-*κ*B (NF-*κ*B), and activating protein-1 (AP-1) (Figure 3). NF-*κ*B activity is repressed by inhibition of p50 and p65 nuclear translocation or by I-*κ*B upregulation, which induces p65 phosphorylation and subsequent proteasomal degradation [72–76]. AP-1 is affected by PPAR*α* through inhibition of its binding to the consensus DNA sequence and by suppressing c-Jun activity [77–79]. Inhibition of inflammatory signaling is important for anticancer therapy in order to reduce mitogenic and angiogenic cytokines and growth factors released by activated immune and stromal cells [80]. Moreover, inhibition of NF-*κ*B, which coordinates a number of antiapoptotic pathways, sensitizes neoplastic cells to nutrient deficiency stress and facilitates apoptosis [81]. NF-*κ*B induces expression of matrix metalloproteinases, such as MMP-9 and urokinase-type plasminogen activator (uPA), and a number of adhesion molecules including ICAM-1, VCAM-1; thus promoting cancer cells’ invasiveness and dissemination [82–84]. Therefore, one could speculate that PPAR*α*- mediated inhibition of NF-*κ*B could contribute to the observed reduction of metastatic spread in melanoma-bearing animals treated with fenofibrate [67].

Recently, a completely new image of PPAR*α* in tumor development has been proposed. Kaipainen and coworkers were the first who initiated studies on the role of PPAR*α* expression in host-tumor interaction. They demonstrated that PPAR*α* depletion in the host significantly reduced tumor growth and metastasis [85]. This effect was not correlated with the tumor type and was independent from the presence or absence of PPAR*α* in the tumor cells. The loss of PPAR*α* in the host was associated instead with decreased microvessel density and enhanced granulocyte infiltration in the tumor tissue and with the elevation of the angiogenesis inhibitor, thrombospondin (TSP-1) [85].

Since necrosis and chronic inflammation within the tumor are associated with intensified macrophage infiltration and poor prognosis [86], it is not entirely clear why granulocyte influx is much more effective in eliminating tumor cells and apparently does not increase the risk of increased tumor vascularization. The possible answer might be a distinct profile of cytokines/chemokines released by macrophages and by granulocytes. The other speculative explanation could be associated with acidic tumor microenvironment, which is known to impair cellular and humoral immune responses. However, it affects differentially macrophages, neutrophils, and lymphocytes, leaving the latter two less prone to this acidic inactivation [87].

## 4. CONCLUDING REMARKS

As presented above, PPAR*α* contributes to the maintenance of physiological homeostasis by multiple mechanisms. Particularly interesting is the interplay between PPAR*α* and AMPK, which represents evolutionary conserved sensor of the metabolic equilibrium, governing the balance between cell death and cell survival. The possible involvement of PPAR*α* in the control of autophagy is an exciting direction to explore, which may reveal new aspects of PPAR*α* role in carcinogenesis.

The metabolic, anti-inflammatory and antiproliferative properties of PPAR*α* ligands provide premises for the potential use as supplementary agents in anticancer treatment, and especially antimetastatic therapies. In addition, low toxicity of synthetic PPAR*α* agonists and the abundance of effective natural ligands provide additional encouragement for the anticancer treatment. However, it should be kept in mind that PPAR*α* was first described to promote peroxisome proliferation and hepatocellular neoplasia in rodents which conversely to humans, and the majority of other species, turned out to be particularly sensitive to PPAR ligands.

Finally, role of PPAR*α* in the tumor-host interactions should be thoroughly studied and explained in order to design effective anticancer therapies with minimized risk of unwanted side effects.

## References

[1] Weinhouse S, Warburg O, Burk D, Schade AL (1956). On respiratory impairment in cancer cells. *Science*.

[2] Warburg O (1956). On the origin of cancer cells. *Science*.

[3] Degenhardt K, Mathew R, Beaudoin B (2006). Autophagy promotes tumor cell survival and restricts necrosis, inflammation, and tumorigenesis. *Cancer Cell*.

[4] Shaw RJ (2006). Glucose metabolism and cancer. *Current Opinion in Cell Biology*.

[5] Ahmed W, Ziouzenkova O, Brown J (2007). PPARs and their metabolic modulation: new mechanisms for transcriptional regulation?. *Journal of Internal Medicine*.

[6] Finck BN, Kelly DP (2002). Peroxisome proliferator-activated receptor *α* (PPAR*α*) signaling in the gene regulatory control of energy metabolism in the normal and diseased heart. *Journal of Molecular and Cellular Cardiology*.

[7] Forman BM, Chen J, Evans RM (1997). Hypolipidemic drugs, polyunsaturated fatty acids, and eicosanoids are ligands for peroxisome proliferator-activated receptors *α* and *δ*. *Proceedings of the National Academy of Sciences of the United States of America*.

[8] Xu HE, Lambert MH, Montana VG (1999). Molecular recognition of fatty acids by peroxisome proliferator-activated receptors. *Molecular Cell*.

[9] Randle PJ (1998). Regulatory interactions between lipids and carbohydrates: the glucose fatty acid cycle after 35 years. *Diabetes/Metabolism Reviews*.

[10] Wolfe RR (1998). Metabolic interactions between glucose and fatty acids in humans. *American Journal of Clinical Nutrition*.

[11] Reddy JK, Hashimoto T (2001). Peroxisomal *β*-oxidation and peroxisome proliferator-activated receptor *α*: an adaptive metabolic system. *Annual Review of Nutrition*.

[12] Alirol E, Martinou JC (2006). Mitochondria and cancer: is there a morphological connection?. *Oncogene*.

[13] Matoba S, Kang J-G, Patino WD (2006). p53 regulates mitochondrial respiration. *Science*.

[14] Brandon M, Baldi P, Wallace DC (2006). Mitochondrial mutations in cancer. *Oncogene*.

[15] Eng C, Kiuru M, Fernandez MJ, Aaltonen LA (2003). A role for mitochondrial enzymes in inherited neoplasia and beyond. *Nature Reviews Cancer*.

[16] Krauss S, Zhang C-Y, Lowell BB (2005). The mitochondrial uncoupling-protein homologues. *Nature Reviews Molecular Cell Biology*.

[17] Gilde AJ, van der Lee KAJM, Willemsen PHM (2003). Peroxisome proliferator-activated receptor (PPAR) *α* and PPAR*β*/*δ*, but not PPAR*γ*, modulate the expression of genes involved in cardiac lipid metabolism. *Circulation Research*.

[18] Yang Q, Li Y (2007). Roles of PPARs on regulating myocardial energy and lipid homeostasis. *Journal of Molecular Medicine*.

[19] Young ME, Patil S, Ying J (2001). Uncoupling protein 3 transcription is regulated by peroxisome proliferator-activated receptor *α* in the adult rodent heart. *The FASEB Journal*.

[20] Pecqueur C, Bui T, Gelly C (2008). Uncoupling protein-2 controls proliferation by promoting fatty acid oxidation and limiting glycolysis-derived pyruvate utilization. *The FASEB Journal*.

[21] Ryu J-W, Hong KH, Maeng JH (2004). Overexpression of uncoupling protein 2 in THP1 monocytes inhibits *β*
_2_ integrin-mediated firm adhesion and transendothelial migration. *Arteriosclerosis, Thrombosis, and Vascular Biology*.

[22] Stavinoha MA, RaySpellicy JW, Essop MF (2004). Evidence for mitochondrial thioesterase 1 as a peroxisome proliferator-activated receptor-*α*-regulated gene in cardiac and skeletal muscle. *American Journal of Physiology*.

[23] Bézaire V, Seifert EL, Harper M-E (2007). Uncoupling protein-3: clues in an ongoing mitochondrial mystery. *The FASEB Journal*.

[24] Himms-Hagen J, Harper M-E (2001). Physiological role of UCP3 may be export of fatty acids from mitochondria when fatty acid oxidation predominates: an hypothesis. *Experimental Biology and Medicine*.

[25] Schrauwen P, Saris WHM, Hesselink MKC (2001). An alternative function for human uncoupling protein 3: protection of mitochondria against accumulation of nonesterified fatty acids inside the mitochondrial matrix. *The FASEB Journal*.

[26] Brand MD, Affourtit C, Esteves TC (2004). Mitochondrial superoxide: production, biological effects, and activation of uncoupling proteins. *Free Radical Biology and Medicine*.

[27] Chen XR, Besson VC, Palmier B, Garcia Y, Plotkine M, Marchand-Leroux C (2007). Neurological recovery-promoting, anti-inflammatory, and anti-oxidative effects afforded by fenofibrate, a PPAR alpha agonist, in traumatic brain injury. *Journal of Neurotrauma*.

[28] Guellich A, Damy T, Lecarpentier Y (2007). Role of oxidative stress in cardiac dysfunction of PPAR*α*
^−/−^ mice. *American Journal of Physiology*.

[29] Du K, Herzig S, Kulkarni RN, Montminy M (2003). TRB3: a tribbles homolog that inhibits Akt/PKB activation by insulin in liver. *Science*.

[30] Rossi S, Graner E, Febbo P (2003). Fatty acid synthase expression defines distinct molecular signatures in prostate cancer. *Molecular Cancer Research*.

[31] Baron A, Migita T, Tang D, Loda M (2004). Fatty acid synthase: a metabolic oncogene in prostate cancer?. *Journal of Cellular Biochemistry*.

[32] Swinnen JV, Van Veldhoven PP, Timmermans L (2003). Fatty acid synthase drives the synthesis of phospholipids partitioning into detergent-resistant membrane microdomains. *Biochemical and Biophysical Research Communications*.

[33] Pizer ES, Thupari J, Han WF (2000). Malonyl-coenzyme-A is a potential mediator of cytotoxicity induced by fatty-acid synthase inhibition in human breast cancer cells and xenografts. *Cancer Research*.

[34] Menendez JA, Lupu R, Colomer R (2005). Targeting fatty acid synthase: potential for therapeutic intervention in Her-2/*neu*-overexpressing breast cancer. *Drug News & Perspectives*.

[35] Menendez JA, Vellon L, Mehmi I (2004). Inhibition of fatty acid synthase (FAS) suppresses *HER2/neu* (*erbB*-2) oncogene overexpression in cancer cells. *Proceedings of the National Academy of Sciences of the United States of America*.

[36] Menendez JA, Vellon L, Oza BP, Lupu R (2005). Does endogenous fatty acid metabolism allow cancer cells to sense hypoxia and mediate hypoxic vasodilatation? Characterization of a novel molecular connection between fatty acid synthase (FAS) and hypoxia-inducible factor-1*α* (HIF-1*α*)-related expression of vascular endothelial growth factor (VEGF) in cancer cells overexpressing Her-2/*neu* oncogene. *Journal of Cellular Biochemistry*.

[37] Botolin D, Wang Y, Christian B, Jump DB (2006). Docosahexaneoic acid (22:6,n-3) regulates rat hepatocyte SREBP-1 nuclear abundance by Erk- and 26S proteasome-dependent pathways. *Journal of Lipid Research*.

[38] Guo Q, Wang P-R, Milot DP (2001). Regulation of lipid metabolism and gene expression by fenofibrate in hamsters. *Biochimica et Biophysica Acta*.

[39] Jump DB, Botolin D, Wang Y, Xu J, Christian B, Demeure O (2005). Fatty acid regulation of hepatic gene transcription. *Journal of Nutrition*.

[40] König B, Koch A, Spielmann J, Hilgenfeld C, Stangl GI, Eder K (2007). Activation of PPAR*α* lowers synthesis and concentration of cholesterol by reduction of nuclear SREBP-2. *Biochemical Pharmacology*.

[41] Srivastava RAK, Jahagirdar R, Azhar S, Sharma S, Bisgaier CL (2006). Peroxisome proliferator-activated receptor-*α* selective ligand reduces adiposity, improves insulin sensitivity and inhibits atherosclerosis in LDL receptor-deficient mice. *Molecular and Cellular Biochemistry*.

[42] Grabacka M, Plonka PM, Urbanska K, Reiss K (2006). Peroxisome proliferator-activated receptor *α* activation decreases metastatic potential of melanoma cells in vitro via down-regulation of Akt. *Clinical Cancer Research*.

[43] McKeown-Eyssen G (1994). Epidemiology of colorectal cancer revisited: are serum triglycerides and/or plasma glucose associated with risk?. *Cancer Epidemiology Biomarkers & Prevention*.

[44] Niho N, Takahashi M, Kitamura T (2003). Concomitant suppression of hyperlipidemia and intestinal polyp formation in *Apc*-deficient mice by peroxisome proliferator-activated receptor ligands. *Cancer Research*.

[45] Buzzai M, Bauer DE, Jones RG (2005). The glucose dependence of Akt-transformed cells can be reversed by pharmacologic activation of fatty acid *β*-oxidation. *Oncogene*.

[46] Jones RG, Plas DR, Kubek S (2005). AMP-activated protein kinase induces a p53-dependent metabolic checkpoint. *Molecular Cell*.

[47] Buzzai M, Jones RG, Amaravadi RK (2007). Systemic treatment with the antidiabetic drug metformin selectively impairs p53-deficient tumor cell growth. *Cancer Research*.

[48] Lee WJ, Kim M, Park H-S (2006). AMPK activation increases fatty acid oxidation in skeletal muscle by activating PPAR*α* and PGC-1. *Biochemical and Biophysical Research Communications*.

[49] Yoon MJ, Lee GY, Chung J-J, Ahn YH, Hong SH, Kim JB (2006). Adiponectin increases fatty acid oxidation in skeletal muscle cells by sequential activation of AMP-activated protein kinase, p38 mitogen-activated protein kinase, and peroxisome proliferator-activated receptor *α*. *Diabetes*.

[50] Elstrom RL, Bauer DE, Buzzai M (2004). Akt stimulates aerobic glycolysis in cancer cells. *Cancer Research*.

[51] Plas DR, Thompson CB (2005). Akt-dependent transformation: there is more to growth than just surviving. *Oncogene*.

[52] Vivanco I, Sawyers CL (2002). The phosphatidylinositol 3-kinase-AKT pathway in human cancer. *Nature Reviews Cancer*.

[53] Garami A, Zwartkruis FJT, Nobukuni T (2003). Insulin activation of Rheb, a mediator of mTOR/S6K/4E-BP signaling, is inhibited by TSC1 and 2. *Molecular Cell*.

[54] Høyer-Hansen M, Jäättelä M (2007). AMP-activated protein kinase: a universal regulator of autophagy?. *Autophagy*.

[55] Zhang Y, Gao X, Saucedo LJ, Ru B, Edgar BA, Pan D (2003). Rheb is a direct target of the tuberous sclerosis tumour suppressor proteins. *Nature Cell Biology*.

[56] Gamerdinger M, Clement AB, Behl C (2007). Cholesterol-like effects of selective cyclooxygenase inhibitors and fibrates on cellular membranes and amyloid-*β* production. *Molecular Pharmacology*.

[57] Suchankova G, Tekle M, Saha AK, Ruderman NB, Clarke SD, Gettys TW (2005). Dietary polyunsaturated fatty acids enhance hepatic AMP-activated protein kinase activity in rats. *Biochemical and Biophysical Research Communications*.

[58] Alessi DR, Sakamoto K, Bayascas JR (2006). LKB1-dependent signaling pathways. *Annual Review of Biochemistry*.

[59] Edinger AL, Thompson CB (2003). Defective autophagy leads to cancer. *Cancer Cell*.

[60] Jin S, DiPaola RS, Mathew R, White E (2007). Metabolic catastrophe as a means to cancer cell death. *Journal of Cell Science*.

[61] Pattingre S, Levine B (2006). Bcl-2 inhibition of autophagy: a new route to cancer?. *Cancer Research*.

[62] Qu X, Yu J, Bhagat G (2003). Promotion of tumorigenesis by heterozygous disruption of the beclin 1 autophagy gene. *Journal of Clinical Investigation*.

[63] Yue Z, Jin S, Yang C, Levine AJ, Heintz N (2003). Beclin 1, an autophagy gene essential for early embryonic development, is a haploinsufficient tumor suppressor. *Proceedings of the National Academy of Sciences of the United States of America*.

[64] Iwamaru A, Kondo Y, Iwado E (2007). Silencing mammalian target of rapamycin signaling by small interfering RNA enhances rapamycin-induced autophagy in malignant glioma cells. *Oncogene*.

[65] Tzatsos A, Tsichlis PN (2007). Energy depletion inhibits phosphatidylinositol 3-kinase/Akt signaling and induces apoptosis via AMP-activated protein kinase-dependent phosphorylation of IRS-1 at Ser-794. *Journal of Biological Chemistry*.

[66] Jin Q, Feng L, Behrens C (2007). Implication of AMP-activated protein kinase and Akt-regulated survivin in lung cancer chemopreventive activities of deguelin. *Cancer Research*.

[67] Grabacka M, Placha W, Plonka PM (2004). Inhibition of melanoma metastases by fenofibrate. *Archives of Dermatological Research*.

[68] Mirouse V, Swick LL, Kazgan N, St Johnston D, Brenman JE (2007). LKB1 and AMPK maintain epithelial cell polarity under energetic stress. *Journal of Cell Biology*.

[69] Bishop-Bailey D (2000). Peroxisome proliferator-activated receptors in the cardiovascular system. *British Journal of Pharmacology*.

[70] Paukkeri E-L, Leppänen T, Sareila O, Vuolteenaho K, Kankaanranta H, Moilanen E (2007). PPAR*α* agonists inhibit nitric oxide production by enhancing iNOS degradation in LPS-treated macrophages. *British Journal of Pharmacology*.

[71] Staels B, Koenig W, Habib A (1998). Activation of human aortic smooth-muscle cells is inhibited by PPAR*α* but not by PPAR*γ* activators. *Nature*.

[72] Cuzzocrea S, Bruscoli S, Mazzon E (2008). Peroxisome proliferator-activated receptor-*α* contributes to the anti-inflammatory activity of glucocorticoids. *Molecular Pharmacology*.

[73] Cuzzocrea S, Mazzon E, Di Paola R (2006). The role of the peroxisome proliferator-activated receptor-*α* (PPAR-*α*) in the regulation of acute inflammation. *Journal of Leukocyte Biology*.

[74] Delerive P, De Bosscher K, Vanden Berghe W, Fruchart J-C, Haegeman G, Staels B (2002). DNA binding-independent induction of I*κ*B*α* gene transcription by PPAR*α*. *Molecular Endocrinology*.

[75] Dubrac S, Stoitzner P, Pirkebner D (2007). Peroxisome proliferator-activated receptor-*α* activation inhibits Langerhans cell function. *Journal of Immunology*.

[76] Vanden Berghe W, Vermeulen L, Delerive P, De Bosscher K, Staels B, Haegeman G (2003). A paradigm for gene regulation: inflammation, NF-*κ*B and PPAR. *Advances in Experimental Medicine and Biology*.

[77] Grau R, Punzón C, Fresno M, Iñiguez MA (2006). Peroxisome-proliferator-activated receptor *α* agonists inhibit cyclo-oxygenase 2 and vascular endothelial growth factor transcriptional activation in human colorectal carcinoma cells via inhibition of activator protein-1. *Biochemical Journal*.

[78] Mishra A, Chaudhary A, Sethi S (2004). Oxidized omega-3 fatty acids inhibit NF-*κ*B activation via a PPAR*α*-dependent pathway. *Arteriosclerosis, Thrombosis, and Vascular Biology*.

[79] Murakami K, Bujo H, Unoki H, Saito Y (2007). Effect of PPAR*α* activation of macrophages on the secretion of inflammatory cytokines in cultured adipocytes. *European Journal of Pharmacology*.

[80] Radisky ES, Radisky DC (2007). Stromal induction of breast cancer: inflammation and invasion. *Reviews in Endocrine & Metabolic Disorders*.

[81] Fabre C, Carvalho G, Tasdemir E (2007). NF-*κ*B inhibition sensitizes to starvation-induced cell death in high-risk myelodysplastic syndrome and acute myeloid leukemia. *Oncogene*.

[82] Aggarwal BB (2004). Nuclear factor-*κ*B: the enemy within. *Cancer Cell*.

[83] Ahn KS, Sethi G, Aggarwal BB (2007). Embelin, an inhibitor of X chromosome-linked inhibitor-of-apoptosis protein, blocks nuclear factor-*κ*B (NF-*κ*B) signaling pathway leading to suppression of NF-*κ*B-regulated antiapoptotic and metastatic gene products. *Molecular Pharmacology*.

[84] Nair AS, Shishodia S, Ahn KS, Kunnumakkara AB, Sethi G, Aggarwal BB (2006). Deguelin, an Akt inhibitor, suppresses I*κ*B*α* kinase activation leading to suppression of NF-*κ*B-regulated gene expression, potentiation of apoptosis, and inhibition of cellular invasion. *Journal of Immunology*.

[85] Kaipainen A, Kieran MW, Huang S (2007). PPAR*α* deficiency in inflammatory cells suppresses tumor growth. *PLoS ONE*.

[86] Mäkitie T, Summanen P, Tarkkanen A, Kivelä T (2001). Tumor-infiltrating macrophages (CD68^+^ cells) and prognosis in malignant uveal melanoma. *Investigative Ophthalmology & Visual Science*.

[87] Lardner A (2001). The effects of extracellular pH on immune function. *Journal of Leukocyte Biology*.

